# Tanshinone IIA mediates SMAD7-YAP interaction to inhibit liver cancer growth by inactivating the transforming growth factor beta signaling pathway

**DOI:** 10.18632/aging.102420

**Published:** 2019-11-11

**Authors:** Lifang Ma, Hongyuan Jiang, Xin Xu, Congcong Zhang, Yongjie Niu, Zhixian Wang, Yuquan Tao, Yan Li, Feng Cai, Xiao Zhang, Xinghe Wang, Yongchun Yu

**Affiliations:** 1 Department of Clinical Laboratory Medicine, Shanghai Municipal Hospital of Traditional Chinese Medicine, Shanghai University of Traditional Chinese Medicine, Shanghai 200071, P.R. China; 2 Institute for Thoracic Oncology, Shanghai Chest Hospital, Shanghai Jiao Tong University, Shanghai 200030, P.R. China; 3 Shanghai Municipal Hospital of Traditional Chinese Medicine, Shanghai University of Traditional Chinese Medicine, Shanghai 200071, P.R. China; 4 Department of Oncology, Shanghai Municipal Hospital of Traditional Chinese Medicine, Shanghai University of Traditional Chinese Medicine, Shanghai 200071, P.R. China; 5 Beijing Shijitan Hospital, Capital Medical University, Beijing 100038, P.R. China

**Keywords:** Tan IIA, TGF-β pathway, SMAD7, YAP, HCC

## Abstract

Tanshinone IIA (TanIIA)-an active constituent of *Salvia miltiorrhiza*, a traditional Chinese medicinal plant-is known to have blood circulation promotion and anti-tumor properties. Tan IIA can induce tumor cell death and inhibit tumor growth. However, the functions and underling molecular mechanisms of Tan IIA action on human liver cancer cells remain poorly understand. In this study, we found that Tanshinone IIA mediates SMAD7-YAP interaction to induce liver cancer cell apoptosis and inhibit cell growth and migration by inactivating the transforming growth factor beta (TGF-β) signaling pathway. Our findings showed that the Tan IIA-SMAD7-YAP regulatory network might be an effective strategy for liver cancer treatment.

## INTRODUCTION

Liver cancer is the fifth most common cancer and the third leading cause of cancer death worldwide. The most common type of primary liver cancer is hepatocellular carcinoma (HCC) [1]. Despite the recent progresses in diagnostic and therapeutic modalities such as surgical treatment, radiofrequency ablation, multi-kinase inhibitor therapy, and liver transplantation, the overall survival and prognosis rates of liver cancer are still unsatisfactory: the 5-year survival rate is only 5%–10% [2]. Therefore, new treatment options for liver cancer are urgently required.

Traditional Chinese medicine is widely used as chemotherapy or adjuvant chemotherapy in liver cancer. It can significantly reduce the clinical symptoms and prolong the survival rate of patients with liver cancer. Tanshinone IIA (Tan IIA), isolated from *Salvia miltiorrhiza*, is an herbal monomer with a specific chemical structure [3]. It exhibits various antitumor effects on esophageal, colorectal, lung, prostate, and gastric cancers by regulating tumor cell growth, apoptosis, invasion, and drug resistance [4–8]. Therefore, Tan IIA might have therapeutic potential in cancer therapy; however, the mechanism by which it inhibits liver cancer growth is not yet known.

The transforming growth factor beta (TGF-β) signaling pathway is involved in many cellular processes, including cell growth, cell differentiation, and apoptosis [9]. Generally, the TGF-β pathway is initiated by the phosphorylation of the cytoplasmic Smad molecules, which results in their translocation to the nucleus where they modulate transcription. TGF-βs are often overexpressed in many disease states such as fibrosis, inflammation, and cancer [10]. They can also facilitate liver fibrosis, cirrhosis, and subsequent progression to HCC [11]. SMAD7 is the negative regulator of the TGF-β signaling pathway and can inhibit it by stably binding to the cytoplasmic domain of activated type I receptors and blocking Smad2/3 phosphorylation [12, 13]. Recently, Sun et al. showed that SMAD7 plays a role of a tumor suppressor gene in liver cancer [11, 12]; however, the mechanism of how SMAD7 suppresses tumor growth in liver cancer remains largely unknown.

The Hippo signaling pathway is a conserved pathway that plays vital roles in controlling liver size and liver tumorigenesis [14]. The Hippo pathway consists of a core kinase cascade, including MAP4K, MST1/2, Sav1, and LATS1/2 as well as transcription co-activators YAP or TAZ, the two major downstream effectors of the Hippo signaling pathway. The loss of tumor suppressor genes and activation of the oncogene YAP or TAZ of the Hippo pathway result in liver tumorigenesis [14–16]. The cross-talk between TGF-β/SMAD and Hippo/YAP signaling pathways have been widely investigated [17]. Makiko et al. showed that TGF-β can synergize with the defects of the Hippo pathway to promote human malignant mesothelioma growth [18]. Alan et al. showed that the components of the Hippo signaling pathway can recruit the nucleosome remodeling and deacetylase complex to inhibit the key genes associated with the TGF-β pathway, thereby indicating whether the TGF-β signaling pathway will support pluripotency or differentiation [19]. Further, TGF-β has been shown to induce Hippo pathway inactivation and promote tumor progression [17]. Taken together, these findings suggest that the cross-talk between the TGF-β/SMAD and Hippo/YAP signaling pathways is critical for tumorigenesis.

In this study, we found that SMAD7 expression was reduced in liver cancer tissues, as well as liver cancer cell lines. The overexpression of SMAD7-Flag could induce cell apoptosis and inhibit cell growth and migration. Further, we found that Tan IIA promoted liver cancer cell apoptosis as well as suppressed cell proliferation, invasion, and migration by up-regulating SMAD7 mRNA and protein expression *in vivo* and *in vitro*. The knockout of SMAD7 can attenuate the ability of Tan IIA to induce apoptosis. Furthermore, we found that SMAD7 and YAP were negatively correlated in liver cancer tissues and cell lines. Moreover, Tan IIA could destabilize the YAP protein. Our findings might form a basis to elucidate a new mechanism of Tan IIA-induced liver cancer cell death.

## RESULTS

### Tan IIA can suppress liver cancer cell growth and induce apoptosis

**Figure 1:**
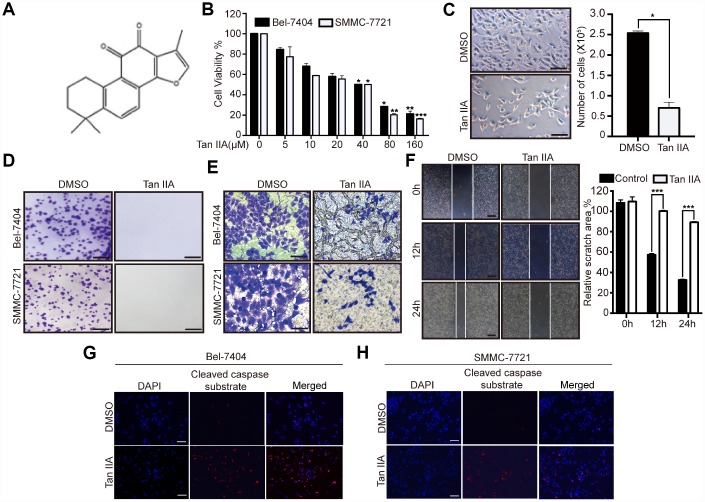
**Tanshinone-IIA (Tan IIA) can inhibit liver cancer cell growth and progression.** (**A**) The Chemical structure of Tan IIA. (**B**) Cell viability of Bel-7404 and SMMC-7721 cells treated with DMSO or dose dependent Tan IIA was determined by CCK-8 cytotoxicity test. *p<0.05, **p<0.01, ***p<0.001 vs DMSO. (**C**) Bright light images of Bel-7404 cells treated with DMSO or 40 μM Tan IIA (left panel). Scale bars: 50μm. Cells were then quantified using ImageJ software and the data are shown as Mean±SD from three independent tests (right panel). *p<0.05. (**D**) Colony formation assay was to determine cell clonogenic ability in Bel-7404 and SMMC-7721 cells with DMSO or 40 μM Tan IIA. Scale bars: 50 μm. (**E**) Cell invasion ability was measured by Transwell invasion assay in Bel-7404 and SMMC-7721 cell lines treated with DMSO or 40 μM Tan IIA. Scale bars: 50 μm. (**F**) Cell wound healing assay was performed to measure cell migration ability in Bel-7404 cells treated with DMSO or 40 μM Tan IIA. The representative images were taken in different time points. Scale bars: 200 μm. ***p<0.001 (**G**, **H**) Tan IIA induced apoptosis marker cleaved caspase substrate expression measured by immunofluorescence assay in Bel-7404 and SMMC-7721 cells. Scale bars: 100 μm.

To determine the anticancer efficiency of Tan IIA (Figure 1A), we treated liver cancer cells Bel-7404, SMMC-7721 and Bel-7402 with serial concentrations of Tan IIA (0, 5, 10, 20, 40, 80, and 160 μM). Tan IIA inhibited the proliferation of Bel-7404, SMMC-7721 and Bel-7402 cells in a dose-dependent manner after 24 h of drug treatment (Figure 1B and Supplementary Figure 1A). Further, when cells were treated with 40 μM of Tan IIA, the cell proliferation activities were 50% lower than those of the DMSO-treated group (Tan IIA 0 μM; Figure 1B, 40 μM*, p*=0.0343<0.05 in Bel-7404, *p*=0.0348<0.05 in SMMC-7721, and *p*=0.035<0.05 in Bel-7402), suggesting that the IC_50_ value of Tan IIA in Bel-7404, SMMC-7721 and Bel-7402 cells was 40 μM. Bright field and cell number images showed that Tan IIA suppressed Bel-7404 cell growth (Figure 1C, *p*=0.0286<0.05). Clone formation assay was also performed to determine the anti-proliferation effect of Tan IIA on liver cancer cells. Tan IIA markedly inhibited liver cancer cell proliferation compared to that of the DMSO group (Figure 1D, and Supplementary Figure 1B).

Subsequently, we determined whether Tan IIA influenced liver cancer cell apoptosis. We performed immunoprecipitation (IP) assay by using stained cleaved caspase substrate; 40 μM Tan IIA induced the expression of the cleaved caspase substrate to promote apoptosis in Bel-7404 and SMMC-7721 cell lines (Figure 1G–1H, and Supplementary Figure 1E). These findings suggested that Tan IIA might play an important role in the tumorigenesis of liver cancer.

### Tan IIA can prevent liver cancer cell progression

To investigate the role of Tan IIA in the progression of liver cancer cells, we performed Transwell invasion and wound healing assays. Bel-7404, SMMC-7721 and Bel-7402 cells treated with 40 μM Tan IIA showed remarkably suppression of cell invasion (Figure 1E, and Supplementary Figure 1C). Similarly, we found that the migration ability of Bel-7404 cells was weakened after treatment with 40 μM Tan IIA (Figure 1F, and Supplementary Figure 1D, *p*<0.001). These results indicated that Tan IIA can inhibit liver cancer cell invasion and migration. Further, it might play an important role in the progression of liver cancer.

### Tan IIA can regulate TGF-β and Hippo/YAP signaling pathways in liver cancer cells

**Figure 2:**
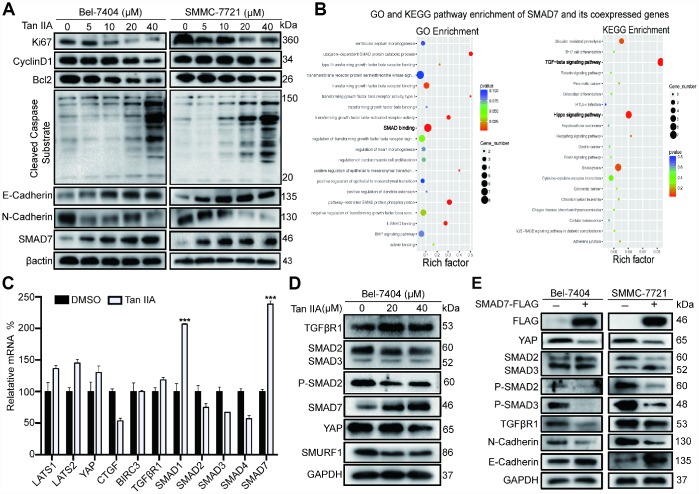
**Tan IIA can regulate TGF-β/SMADs and Hippo/YAP signaling pathway in liver cancer cells.** (**A**) Bel-7404 and SMMC-7721 cells were cultured with DMSO or dose dependent Tan IIA (5, 10, 20, 40 μM) for 24 h and protein expression levels of Ki67, CyclinD1, Bcl2, Cleaved Caspase Substrate, E-cadherin and N-cadherin were measured by western blot assay. (**B**) GO biological process and KEGG pathway enrichment of SMAD7 co-expressed top 50 genes from cBioPortal database. (**C**) TGF-β/SMADs and Hippo/YAP signaling pathway related genes were determined by real-time quantitative PCR assay with DMSO or Tan IIA (40 μM). (**D**) The protein expression level of TGF-β/SMADs components TGFβR1, SMAD2, SMAD3, P-SMAD2, SMAD7 and Hippo pathway effector YAP and E3 ligase SMURF1, βTrcp were analyzed by western blot assay with DMSO, 20 μM, 40 μM Tan IIA in Bel-7404 cells. (**E**) Indicated protein expression levels were measured by western blot assay with or without SMAD7-Flag overexpression in Bel-7404 and SMMC-7721 cells.

**Figure 3:**
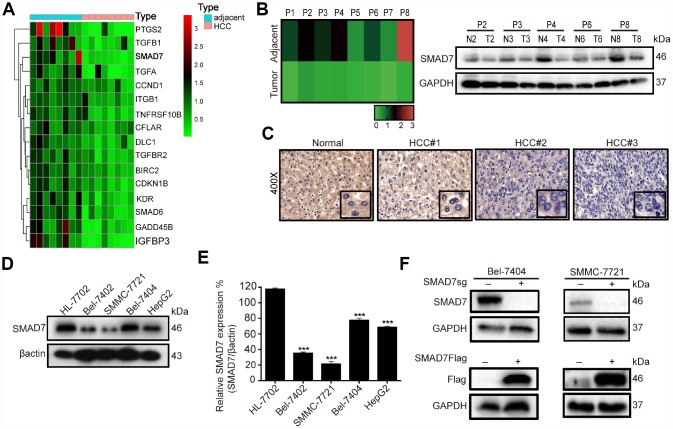
**SMAD7 is downregulated in liver cancer tissues and cell lines.** (**A**) Microarray analysis of gene expression from RNA extracted in 8 paired human liver cancer tissues and matched normal liver tissues. (**B**) RT-qPCR assay detected the mRNA expression level of SMAD7 in 8 paired human liver cancer tissues (left panel). Western blot assay detected the protein expression level of SMAD7 in 5 paired liver cancer tissues which SMAD7 were differentially expressed (right panel). (**C**) Representative IHC images of SMAD7 staining in normal liver tissues and liver cancer tissues at 400Χ magnifications. (**D**) The SMAD7 protein level was analyzed in normal human liver cell line HL-7702 and liver cancer cell lines Bel-7402, SMMC-7721, Bel-7404 and HepG2 by western bolt assay. β-actin served as loading control. (**E**) Relative SMAD7 protein expression was calculated by the ration of SMAD7/β-actin in different cell lines from three independent tests. ***p<0.001 vs HL-7702. (**F**) The protein expression of SMAD7 in Bel-7404 and SMMC-7721 were analyzed by western blot with or without SMAD7-Flag or SMAD7-sgRNA virus infection. β-actin served as loading control.

To validate the anti-tumor efficiency of Tan IIA at the molecular level, we performed western blot (WB) assay to detect the protein expression level of proliferation, apoptosis, and epithelial to mesenchymal transition (EMT) markers. The proliferation, cell cycle, and apoptosis inhibitor markers Ki67, CyclinD1, and Bcl2 respectively were significantly decreased in Bel-7404, SMMC-7721 and Bel-7402 cells after treatment with 40 μM Tan IIA (Figure 2A, and Supplementary Figure 2A). In contrast, the pro-apoptotic marker cleaved caspase substrate was up-regulated in cells treated with 40 μM Tan IIA (Figure 2A, and Supplementary Figure 2A). Further, the EMT marker E-cadherin level gradually increased, whereas the N-cadherin level dose-dependently decreased after treatment with Tan IIA. Surprisingly, we found that the TGF-β component SMAD7 was up-regulated in the Tan IIA group (Figure 2A, and Supplementary Figure 2A), which was consistent with the anti-tumor effect noted in our PCR array results (Figure 3A).

As SMAD7 was downregulated in liver cancer and was associated with malignant disease [11, 12], we explored its role as a cancer suppressor in liver cancer. We selected top 50 genes co-expressed with SMAD7 from the cBioPortal database and subjected them to KEGG pathway analysis and GO biological process analysis. As shown in the bubble diagram, the top 50 co-expressed genes participated in SMAD binding in the TGF-β and Hippo signaling pathways (Figure 2B). These results suggested that the proteins interacting with SMAD7 might play a critical role in the TGF-β and Hippo signaling pathway. Next, we performed qRT-PCR and WB assay to determine whether Tan IIA can regulate TGF-β and Hippo signaling pathways. We found that Tan IIA up-regulated SMAD7 and SMAD1 mRNA expression, but did not affect the Hippo pathway components (Figure 2C). We also found that Tan IIA down-regulated the protein levels of SMAD2, SMAD3, P-SMAD2, YAP, and SMURF1 and up-regulated the expression of SMAD7 (Figure 2D). Next, we overexpressed SMAD7-Flag in Bel-7404 and SMMC-7721 cell lines and performed WB assay, which also yielded the same results as that after treatment with Tan IIA (Figure 2E). These results suggest that Tan IIA might exhibit its anti-tumor activity by up-regulating the expression level of SMAD7 in a TGF-β- and Hippo/YAP signaling pathway-dependent manner.

### SMAD7 was down-regulated in liver cancer tissues and cells

To explore the role of liver cancer-related genes in tumorigenesis, we performed medium-throughput PCR assay to detect the mRNA expression level of these genes in human liver cancer tissues and found a series of differentially expressed genes, including SMAD7 (Figure 3A, Supplementary Table 1). To investigate the biological function of SMAD7 in liver cancer, we initially re-measured the relative mRNA expression of SMAD7 in 8 paired liver cancer tissues (Figure 3B, left panel). Next, we selected 5 paired tissues in which SMAD7 was largely down-regulated to detect its protein level. We found that SMAD7 was down-regulated at the mRNA and protein levels (Figure 3B, right panel). We also performed immunohistochemistry (IHC) to detect SMAD7 staining in normal and liver cancer tissues. SMAD7 expression was higher in normal tissues than in liver cancer tissues (Figure 3C), suggesting that the down-regulation of SMAD7 was associated with liver cancer progression.

Subsequently, we detected SMAD7 protein expression in 4 human liver cancer cell lines and in the normal liver epithelial cell line HL-7702. We found that the expression level of SMAD7 was lower in the liver cancer cell lines than in HL-7702 (Figure 3D–3E, *p*<0.001). We selected Bel-7404 and SMMC-7721 cell lines for the subsequent experiment and confirmed the overexpression and knockout efficiency of SMAD7 in these two cell lines (Figure 3F). The findings revealed that SMAD7 was down-regulated in liver cancer tissues and cell lines and might play a critical role in the development and progression of liver cancer.

### Overexpression of SMAD7 suppresses liver cancer cell growth and promotes apoptosis *in vitro* and *in vivo*

**Figure 4:**
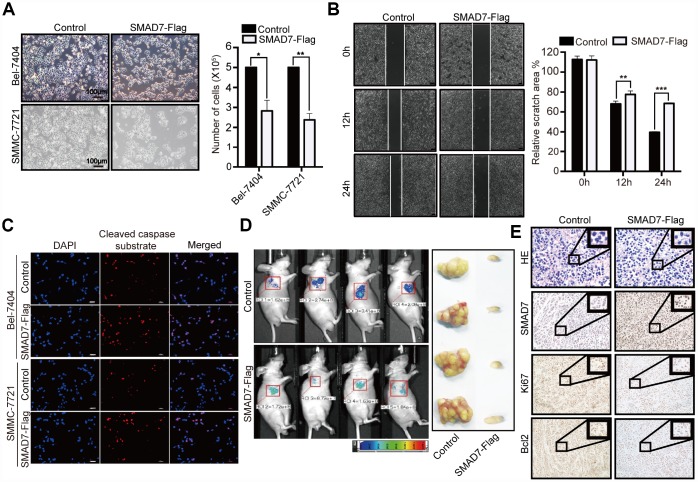
**SMAD7 can inhibit liver cancer growth and migration.** (**A**) The bright images of Bel-7404 and SMMC-7721 cells expressing GFP (Control) or SMAD7-Flag. Cells were plated at a density of 5,000 per well and imaged 7 days later (left panel). Scale bar: 100 μm. Cells were then quantified using ImageJ software and the relative cell number are shown as Mean ± SD from three independent tests (right panel). *p<0.05, **p<0.01 vs control. (**B**) Bel-7404 cells were infected with control or SMAD7-Flag virus as indicated, and cell migration ability was assessed by cell scratch assay. The images are taken at the 0 h, 12 h and 24 h time points after the cultures were wounded by scratching (left panel). Quantification of relative scratch area is measured by ImageJ software, datas are showed as Mean ± SD of three independent experiments (right panel). **p<0.01, ***p<0.001. (**C**) SMAD7-Flag induced apoptosis marker cleaved caspase substrate expression measured by immunofluorescence assay in Bel-7404 and SMMC-7721 cells. Scale bars: 100 μm. (**D**) Representative images of control (expressing GFP tag) and SMAD7-Flag (expressing GFP tag) Xenografts mouse were analyzed by small animals *in vivo* imaging system (left panel) and representative images of tumor as indicated (right panel). (**E**) Representative HE and IHC images of SMAD7, proliferation marker Ki67 and apoptosis marker Bcl2 staining were taken in control and SMAD7-Flag Xenografts mouse tissue groups at 400Χ magnifications.

To further determine the biological role of SMAD7 in liver cancer cells, we investigated the effect of the overexpression of SMAD7-Flag and found that this markedly decreased the cell growth ability compared with that of the control in Bel-7404 and SMMC-7721 cells (Figure 4A, *p=0.0110<0.05, **p=0.0026<0.01). Furthermore, we found that the overexpression of SMAD7 impaired the migration ability of liver cancer cells (Figure 4B, **p=0.0021<0.01, ***p<0.001) and enhanced their apoptosis rate, which was indicated using the cleaved caspase substrate (Figure 4C). Taken together, these findings suggested that the overexpression of SMAD7 might prevent liver cancer development *in vitro*.

Next, we explored the biological role of SMAD7 *in vivo*; the tumor volume was reduced in SMAD7-Flag overexpression group compared to that in the control group (expressing GFP tag) in the xenograft mouse model (Figure 4D). Next, we performed IHC assay to detect the cell proliferation and apoptosis marker Ki67 and Bcl2 levels in SMAD7-Flag overexpression and control groups and found that their levels were markedly decreased in the SMAD7-Flag groups (Figure 4E). Taken together, these data indicated that SMAD7 is important for inhibiting human liver cancer cell growth and migration and might play a role of a tumor suppressor gene in liver cancer.

### SMAD7 is involved in Tan IIA-mediated inhibition of liver cancer tumorigenesis

**Figure 5:**
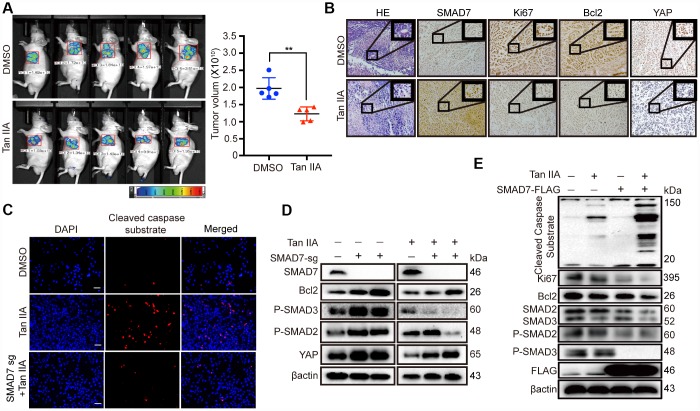
**Tan IIA can suppress liver cancer cell growth with a TGF-β dependent manner and partially through up-regulating SMAD7.** (**A**) The representative images of DMSO and Tan IIA groups were analyzed by small animals *in vivo* imaging system. Two groups were seeded with 5**×**10^6^ Bel-7404 cells and then 14 days later injected with diluted DMSO or Tan IIA (10mg/kg/d) resolution. Tumor images and volumes were taken and measured in 20 days after drugs injection. n =5 per group. **p < 0.01. (**B**) Representative HE and IHC pictures of SMAD7, Ki67, Bcl2 and YAP staining in DMSO and Tan IIA Xenografts mouse tissues at 400Χ magnifications. (**C**) Cleaved caspase substrate was detected by immunofluorescence assay in DMSO, Tan IIA (40 μM) and Tan IIA along with SMAD7 knockout groups for 24 h. Scale bar: 100 μm. (**D**, **E**) The protein expression levels were detected by western blot assay in indicated groups.

To validate the results of the anti-tumor effect of Tan IIA in the *in vitro* anti-tumorigenesis assays, we generated a xenograft mouse model in Balb/c nude mice by using Bel-7404 cells expressing a control GFP tag. We found that the GFP tumor region was considerably smaller in the Tan IIA-treated group than in the DMSO-treated group (Figure 5A, left panel). The tumor volume of the Tan IIA-treated groups was smaller than that of the DMSO-treated groups (Figure 5A, right panel, **p=0.0021<0.01). To further investigate the proliferation ability and apoptosis level *in vivo*, we performed IHC assay to detect the level of the proliferation marker Ki67 and apoptosis marker Bcl2. Tan IIA decreased the protein expression of Ki67 and Bcl2 and promoted SMAD7 expression *in vivo* (Figure 5B). Further, the oncogene YAP, which was predicted to interact with SMAD7, was remarkably reduced in the Tan IIA-treated group (Figure 5B).

Subsequently, to investigate whether SMAD7 is essential in Tan IIA-mediated apoptosis in liver cancer cell lines, we performed IF analysis to test the level of the apoptosis marker cleaved caspase substrate and found that SMAD7 knockout can impair the apoptosis-inducing ability of Tan IIA in Bel-7404 cells (Figure 5C). We also performed WB rescue assay to detect the role of SMAD7 in the apoptosis-inducing ability of Tan IIA. We found that Bcl2, P-SMAD2, P-SMAD3, and YAP were down-regulated in the Tan IIA group. Nevertheless, the knockout of SMAD7 largely rescued their protein expression levels. Two independent stable SMAD7 knockout cell lines treated with Tan IIA partially rescued the Tan IIA-induced apoptosis and inhibited YAP expression (Figure 5D).

Because SMAD7 expression is less in liver cancer cells, detecting the protein level of SMAD7 was difficult. Therefore, we also performed SMAD7-Flag overexpression assay and found that cells treated with Tan IIA simultaneously with SMAD7 overexpression could largely inhibit the TGF-β/SMADs signaling pathway and induce apoptosis (Figure 5E). Taken together, these results strongly suggest that SMAD7 is involved in Tan IIA-induced liver cancer apoptosis *in vivo* and *in vitro*.

### SMAD7 and YAP can interact with each other in liver cancer

**Figure 6:**
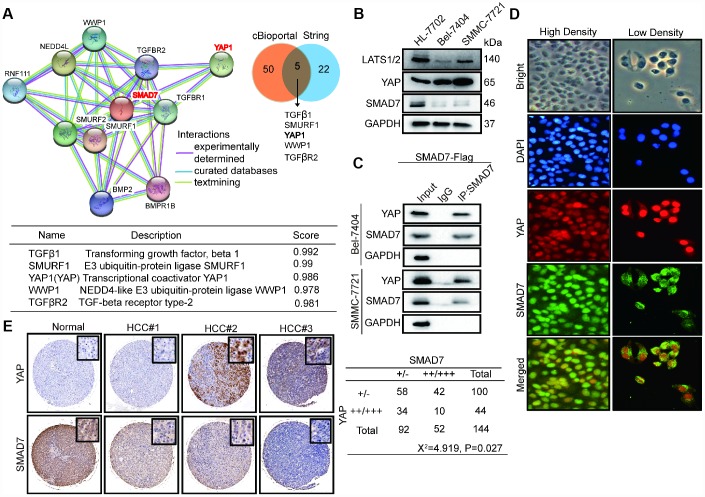
**SMAD7 and YAP can interact with each other and negatively correlate in liver cancer.** (**A**) The protein-protein network shows SMAD7 related top 10 genes which were obtained from string database (left panel). Venn diagram showing overlapping of SMAD7 associated genes in cBioPortal and string databases (right panel). The detail information of overlapping 5 genes (lower panel). (**B**) The protein expression level of LATS1/2, YAP and SMAD7 were measured by western blot assay in normal liver cells and liver cancer cell lines. (**C**) SMAD7 binds to endogenous YAP which measured by co-immunoprecipitation assay in SMAD7-Flag over-expressed Bel-7404 and SMMC-7721 stable cell lines. (**D**) YAP and SMAD7 intracellular localization in Bel-7404 cells in low cell density and high cell density. (**E**) Representative IHC pictures of SMAD7 and YAP staining showed protein expression level and location in normal and HCC tissues, and the correlated levels of SMAD7 and YAP expression. Statistical analysis of the TMA data is shown in the bottom panel.

We analyzed the expression of SMAD7 co-expressed proteins from the cBioPortal database and performed GO biological function and KEGG signaling pathway analyses (Figure 2B). To validate the accuracy, we used SMAD7 interacting proteins from the String database and selected the top 22 proteins and the intersection with the top 50 proteins from the cBioPortal database. The Venn diagram showed that genes associated with SMAD7 that were common in cBioPortal and string databases (Figure 6A, right panel) were *TGFβ1*, *SMURF1*, *YAP*, *WWP1*, and *TGFβR2* (Figure 6A, lower panel). The protein–protein networks showed the top 10 SMAD7-related genes were obtained from the string database (Figure 6A, left panel). Based on these results and our findings from previous several studies focusing on the YAP-mediated mechanism of liver cancer development promotion [20–22], we investigated whether YAP and SMAD7 can interact with each other and determined their roles in Tan IIA-induced antitumor activity.

First, we detected the protein level of YAP and SMAD7 in HL-7702, Bel-7404, and SMMC-7721 cell lines. The expression of SMAD7 and LATS1/2 was down-regulated in liver cancer cells. However, YAP was abundantly expressed in Bel-7404 and SMMC-7721 cells (Figure 6B). Next, CP assay revealed that SMAD7 can bind to endogenous YAP in the liver cancer cell lines overexpressing SMAD7-Flag (Figure 6C). The Hippo/YAP pathway has been shown to act in a cell density-dependent manner: it remains active when the cell density is the highest and inactive when the cell density is lower [23]. Our results showed that SMAD7 remained in the cytoplasm under low cell density conditions, whereas it could translocate into the nucleus and interact with YAP under high cell density condition (Figure 6D).

### SMAD7 and YAP are negatively correlated in liver cancer

To further reveal the relationship between YAP and SMAD7, we performed the IHC assay by using TMA- containing human liver cancer samples. We found that SMAD7 expression was down-regulated in HCC tissues, and YAP proteins were highly expressed in a subset of human liver cancers and negatively correlated with each other (Figure 6E).

Next, we analyzed the correlation between the expression level of SMAD7 and the clinical features of patients with liver cancer. Based on the clinical information for TMA (provided by the U.S. Biomax), we found a negative correlation between tumor grade and SMAD7 expression (*p*=0.013<0.05), indicating that patients with high-grade liver cancer have lower SMAD7 expression level (Table 1), whereas no significant correlation was found between tumor stage and the expression of SMAD7 expression (*p*=0.59>0.05; Table 1). Taken together, these results reveal a close relationship between SMAD7 and YAP in liver cancer.

**Table 1:** Correlation of SMAD7 protein expression with clinicopathologic features.

**Clinicopathologic**	**Cases(N)**	**SMAD7 expression**		***p***
**features**		**Low**	**High**	
**Ages**				
≥60	30	22	8	0.207
<60	110	66	44	
**Gender**				
Male	104	59	45	0.116
Female	36	26	10	
**Grade**				
1	21	6	15	**0.013**
2	109	69	40	
3	10	6	4	
**Stage**				
I	8	4	4	0.59
II	50	30	20	
III	82	54	28	
**T Classification**				
T1	10	4	6	0.853
T2	61	30	31	
T3	69	34	35	

### Tan IIA destabilized YAP by regulating SMAD7 in liver cancer

**Figure 7:**
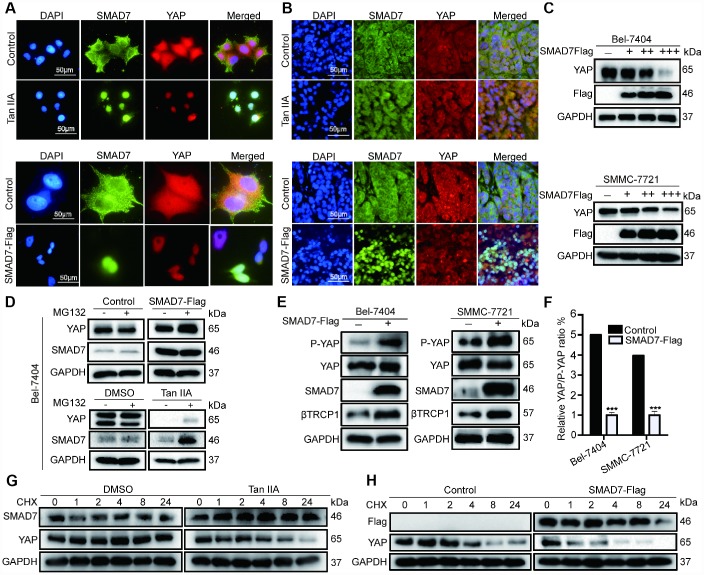
**Tan IIA destabilized YAP through regulating SMAD7 in liver cancer.** (**A**, **B**) Colocalization of SMAD7 and YAP in Bel-7404 cells and xenograft tissues analyzing by immunofluorescence assay. Scale bar: 50 μm. (**C**) Western blot assay analysis of SAMD7 and YAP protein expression in Bel-7404 and SMMC-7721 cells transfected with different dose SMAD7-Flag. (**D**) YAP and SMAD7 protein expression level were analyzed by western blot assay treated with or without MG132 in different groups. (**E**) P-YAP, YAP and βTrcp1 protein expression level were measured by western blot assay with or without SMAD7-Flag overexpression in Bel-7404 and SMMC-7721 cells. (**F**) Relative YAP/P-YAP ratio was measured by gray value. ***p<0.001 vs control. (**G**, **H**) Bel-7404 cells were stably transfected with or without SMAD7-Flag, with or without Tan IIA treated, and protein synthesis was blocked by treatment of CHX for the different time points. Relative SMAD7 and YAP protein levels were detected by western blot assay.

To test whether Tan IIA and SMAD7 have the same effect on YAP localization *in vitro* and *in vivo*, we performed IP assay to detect the co-localization of SMAD7 and YAP in cells and tissues. We found that cells treated with Tan IIA and showing SMAD7-Flag overexpression became small and had shrunken nuclei (Figure 7A–7B). Thus, Tan IIA and SMAD7 might promote cell apoptosis. YAP showed reduced expression and was co-localized with SMAD7 in the Tan IIA group and SMAD7-Flag overexpression group (Figure 7A–7B). Taken together, our results suggested that Tan IIA can promote SMAD7 expression and nuclear transport. Further, binding to YAP promoted the translocation of YAP into the cytoplasm. To test our hypothesis, we transfected Bel-7404 and SMMC-7721 cells with 1, 2, and 3 μg of SMAD-Flag plasmid. The results showed that 3 μg SMAD7-Flag prominently decreased the expression of YAP (Figure 7C).

Subsequently, to confirm how SAMD7 and Tan IIA can regulate YAP, we treated cells with the proteasome inhibitor MG132 and protein synthesis inhibitor CHX. We found that the expression level of YAP and SMAD7 changed slightly in the control, SMAD7-Flag, DMSO, and Tan IIA groups with or without MG132 treatment (Figure 7D). Therefore, we concluded that Tan IIA and SMAD7 can regulate YAP at the protein level. Next, we performed WB assay to detect the degradation of YAP in control, SMAD7-Flag-treated, DMSO-treated, and Tan IIA with CHX–treated cells. We found that YAP protein was easily degraded in the Tan IIA- or SMAD7-Flag-treated groups (Figure 7G–7H). These findings suggested that Tan IIA partially regulated YAP protein stabilization by upregulating SMAD7.

Our previous studies showed that E3 ligase βTrcp can bind to YAP and promote its degradation. Therefore, we detected the expression of βTrcp, YAP, P-YAP, and SMAD7 with or without SMAD7-Flag overexpression in Bel-7404 and SMMC-7721 cell lines. The overexpression of SMAD7-Flag was found to largely up-regulate βTrcp and P-YAP expression (Figure 7E, and Supplementary Figure 2B), whereas the YAP/P-YAP ratio was decreased in the SMAD7-Flag group (Figure 7F, ***p=0.0002<0.01). Taken together, our results suggest that Tan IIA can upregulate SMAD7 to promote E3 ligase βTrcp expression that can promote YAP protein degradation in liver cancer.

## DISCUSSION

To our knowledge, this is the first study to show that the Tan IIA-SMAD7-YAP regulatory network is a novel strategy for liver cancer treatment. Tan IIA is a natural flavonoid isolated from *Salvia miltiorrhiza*; it can inhibit liver cancer growth *in vivo* and *in vitro*. Tan IIA directly suppressed liver cancer cell proliferation, migration, and invasion as well as induced apoptosis (Figure 1B–1H, 2A, and Supplementary Figures 1A–1E, and 2A). We also found that Tan IIA up-regulated both the mRNA and protein expression of SMAD7, which was identified as a downstream target of Tan IIA in a TGF-β/SMAD and Hippo/YAP signaling pathway-dependent manner (Figure 2C–2D). The knockout of SMAD7 partially rescued the Tan IIA-induced cell apoptosis (Figure 5C). Considering the crucial anti-tumor function of Tan IIA in liver cancer, our study showed the therapeutic importance of Tan IIA and SMAD7 in this malignancy. Taken together, our findings might provide new perspectives and treatment methods on the management of patients with liver cancer.

SMAD7, a TGF-β target gene, acts as a physiological feedback inhibitor of TGF-β family cytokines [24]. Accordingly, SMAD7 might acts as both oncogene and tumor suppressor gene, which depends on the spatial–temporal regulation of its biological action. It was found to have not only tumor promotive but also suppressive functions in different kinds of cancer [25–27]. Indeed, it has been reported to be a controversial effecter in the development of several cancers. However, recent studies have revealed new mechanistic insights into the tumor-suppressive functions of SMAD7 in hepatocarcinogenesis and subsequent tumor progression [11, 12]. In the present study, we found that the mRNA and protein expression of SMAD7 in liver cancer tissues was significantly down-regulated compared to that in the adjacent normal tissues (Figure 3A–3C), as well as in liver cancer cell lines (Figure 3D–3E). We also found that low SMAD7 protein expression was associated with tumor grade (Table 1): when SMAD7 expression was lower, the cancer grade was higher. Taken together, our findings suggest that SMAD7 might exhibit tumor suppressor function, and the lower expression of SMAD7 in liver cancers might predict poor outcome. The overexpression of SMAD7-Flag in liver cancer cells largely induced cell apoptosis and inhibited cell growth and migration *in vitro* and *in vivo* (Figure 4A–4E). Therefore, Tan IIA might exert its antitumor activity by upregulating SMAD7.

To better understand the biological function of SMAD7 in inhibiting liver cancer development and progression, we generated a protein–protein interaction network for SAMD7 by using the cBioPortal database. We selected 50 genes co-expressed with SMAD7 and subjected them to KEGG pathway analysis and GO biological process analysis. As show in the bubble diagram, the top 50 co-expressed genes participated in SMAD binding as well as TGF-β and Hippo signaling pathway (Figure 2B). Subsequently, the top 22 genes from String database that interacted with SMAD7 were intersected with the top 50 genes from cBioPortal database. In all, 5 overlapping genes, i.e., TGFβ1, SMURF1, YAP1, WWP1, and TGFβR2, were identified (Figure 5A). Because the cross-talk between the TGF-β/SMADs and Hippo/YAP signaling pathway has been widely studied [17–19], and, based on the results of our present study and previous several studies that focus on the mechanism of YAP-mediated promotion of liver cancer development [20, 21], we selected the Hippo pathway effector YAP to determine the role of Tan IIA in the regulation of SMAD7 expression in liver cancer.

We found that SMAD7 and YAP were negatively correlated in liver cancer tissues and cell lines (Figure 6B, 6E). Further, SMAD7 could be translocated into the nucleus to bind to YAP (Figure 6D) under high-cell-density condition, during which the Hippo pathway is active. Next, we intended to determine whether the anti-tumor activity of Tan IIA exerted by up-regulating SMAD7 is dependent on YAP. Our results showed that Tan IIA and SMAD7 can both down-regulate YAP expression via protein degradation processes (Figure 7D, 7G, 7H), which might depend on E3 ligase βTrcp (Figure 7E, and Supplementary Figure 2B), because several studies have revealed that E3 ligase βTrcp can target YAP to promote protein degradation [28–30].

Tan IIA has been widely used for treating cardiovascular diseases and is believed to have antitumor effects [6, 31]. Recent studies have revealed the inhibitory effect of Tan IIA on cell proliferation and apoptosis promotion in several types of cancers such as hepatocellular carcinoma, gastric carcinoma, and colorectal carcinoma [3, 7, 32]. Therefore, it serves as an antitumor monomer of traditional Chinese medicine with promising investigation potential. Our findings suggested that Tan IIA can significantly inhibit liver cancer cell growth and migration *in vivo* and *in vitro* by regulating SMAD7-YAP expression in a TGF-β/SMAD signaling pathway-dependent manner.

Taken together, our findings suggested that Tan IIA can suppress liver cancer cell growth, migration, and invasion as well as promote cell apoptosis; it plays a role as an antitumor agent by regulating the expression of SMAD7-YAP. Mechanistically, our study might provide a new effective targeted therapeutic strategy for the treatment of liver cancer. Further, our findings support the use of Tan IIA as an antitumor drug owing to its ability to induce the expression of SMAD7.

In conclusion, to our knowledge, this is the first study to show that Tan IIA can inhibit liver cancer proliferation, migration, and invasion as well as promote cell apoptosis by regulating the expression of SMAD7 and YAP in a TGFβ signaling pathway-dependent manner.

## MATERIALS AND METHODS

### Human liver cancer samples

Matched pairs of liver cancer and adjacent non-tumor tissues from eight patients included in our previous studies were obtained [33]. The 8 patients with HCC were included 4 males and 4 females, aged from 30 to 70 years, staged from I to III. In brief, the tissues were immediately frozen in liquid nitrogen after surgery. None of the patients received radiotherapy, chemotherapy, or other treatments before surgery. All patients had consented to the use of samples and provided written informed consent during their hospitalization. The informed consent documents were reviewed and approved by the Ethics Committee of the Shanghai Municipal Hospital of Traditional Chinese Medicine.

### Polymerase chain reaction array

For eight paired liver cancer tissues, total RNA was extracted using the RNeasy Plus Mini Kit (QIAGEN, Hilden, Germany). Gene expression profiles were analyzed using the polymerase chain reaction (PCR) array for human liver cancer-associated genes, according to manufacturer’s protocol (Wcgene Biotech, Shanghai, China). Data were analyzed using Graphpad prism7 software. The genes with biological significance are listed in Supplementary Table 1.

### Cell culture, vectors, and drug

HL-7702, Bel-7402, Bel-7404, SMMC-7721, HepG2, and HEK-293T cells were cultured in Dulbecco’s Modified Eagle’s Medium (DMEM) supplemented with 10% fetal bovine serum (FBS) and 1% Penicillin–Streptomycin.

Small guide RNAs against SMAD7 were cloned into LentiCrispr v2 vectors by using the primers listed in Supplementary Table 2. SMAD7-Flag was amplified from corresponding cDNA purchased from Origene (Beijing, China) by using PCR and subcloned into PGIPZ2a by using the primers listed in Supplementary Table 2.

Tan IIA was purchased from TAUTO Biotech (Shanghai, China) and dissolved in DMSO. The cells were treated with Tan IIA (0-160 μM), MG132 (25 μM; Selleck, Huston, USA), or cycloheximide (CHX; 50 μg/ml; Sigma) for 24 h before harvest.

### Immunohistochemistry, immunofluorescence, and western blotting

For immunohistochemistry (IHC), human liver cancer tissue microarray (TMA) slides were purchased from U.S. Biomax (Rockville, MD, USA). Mouse tumor tissues were processed for paraffin sectioning and mounted on slides. The slides were incubated in primary antibodies against SMAD7 (1:200, R&D Systems, #MAB2029), Bcl2 (1:200, ImmunoWay, #YM3041), Ki67 (1:200, ImmunoWay, #YT2467), and YAP (1:200, CST, #12395), and then incubated overnight at 4°C.

For immunofluorescence (IF) study, cells were cultured in a 24-well plate with a coverslip, treated with DMSO or Tan IIA for 24 h, and then fixed with 4% paraformaldehyde for 15 min. Subsequently, they were washed three times with phosphate buffered saline (PBS) and incubated with blocking buffer (1% FBS + 1% goat serum + 0.1% Triton X-100), and then incubated overnight at 4°C in primary antibodies against cleaved caspase substrate (1:200, CST, #8698), SMAD7, YAP (1:200, Abcam, #ab52771), and Cy3 or FITC fluorescent-conjugated secondary antibodies (1: 300, Servicebio, #GB22301, #GB22303, #GB21301, and #GB21303).

For western blot (WB), the protein levels were determined using primary antibodies against Flag (CST, #2368), Ki67, CyclinD1(1:2000, CST, #2352), Bcl2, cleaved caspase substrate, TGFβR1 (1:2000, ImmunoWay, #YT4627), SMAD2/3(1:2000, ImmunoWay, #YT4332), P-SMAD2 (1:2000, ImmunoWay, #YP0840), P-SMAD3(1:2000, Abcam, #ab52903), SMAD7, GAPDH (1:2000, ImmunoWay, #YM3445), β-actin (1:2000, ImmunoWay, #YM3028), E-cadherin (1:2000, ImmunoWay, #YT1454), N-cadherin (1:2000, ImmunoWay, #YT2988), YAP, Smurf1 (1:2000, Abcam, #ab57573), Lats1/2 (1:2000, Abcam, #ab70565), βTrcp(1:2000, CST, #4394), and horseradish peroxidase-linked secondary antibodies (1:2000, CST, #7076 and #7074).

### Immunoprecipitation

Cells were washed with PBS for three times and subsequently lysed in WB or immunoprecipitation (IP) lysis buffer (Beyotime, Jiangsu, China). Protein lysates were lysed for 30 min on ice and then centrifuged at 12,000×*g* for 10 min to collect proteins. After preclearing for 1 h with 50 μl of protein A/G-Sepharose (Life Technologies Corporation, Carlsbad, CA), the supernatants were incubated at 4°C overnight with 3 μg SMAD7 antibody crosslinked to protein A/G-Sepharose beads. Beads were washed five times with lysis buffer, resuspended in SDS loading buffer, and analyzed using WB by using antibodies as indicated.

### CCK8 and clone formation assay

Cell proliferation was measured using a CCK8-based proliferation assay. Bel-7404 and SMMC-7721 cells (0.8×10^4^ cells in 96-well culture plates) were treated with DMSO or Tan IIA (5, 10, 20, 40, 80, and 160 μM) for 24 h. The medium was incubated with 1:10 volume of CCK-8 solution for 1–4 h in an incubator with 5% CO_2_ at 37°C. Absorbance was read at 450 nm on a microplate reader. Cell viability (%) was calculated as (experimental absorbance-background absorbance)/ (control absorbance - background absorbance) ×100%.

Clone formation assay: Liver cancer cells were seeded with 600 cells/well in 12-well plates, and then treated with DMSO or Tan IIA (40 μM) when the cells adhered. The fresh medium was replaced every three days. After 7-10 days of incubation, cell colonies consisting of more than 50 cells stained with crystal violet were counted, and pictures were obtained.

### Cell migration and invasion assay

Cell migration ability was measured using the wound healing assay, which has been described in our previous studies [34].

Transwell assay was performed to measure the cell invasion activity. In brief, a Transwell chamber was placed in a 24-well plate. Next, 100 μl diluted matrigel was added in the upper chamber and the plate was placed in an incubator with 5% CO_2_ at 37°C for at least 1 h. The remaining liquid was carefully removed from the Transwell insert. Cells were collected and suspended in serum-free DMEM containing 0.1% BSA. Subsequently, cells (5×10^4^/200μl) were seeded in the Transwell upper chambers, and 500 μl complete medium containing 20% FBS was added to the 24-well plate placed in an incubator with 5% CO_2_ at 37°C for 24 h. The cells on the upper surface of the filter were completely removed. Cells on the lower surface of the membrane were fixed with 95% ethanol for 10–15 min, stained with 0.1% crystal blue solution for 10 min, washed with PBS for several times, and then dried. Finally, the cell numbers were counted, and pictures were obtained under a microscope.

### Quantitative real time-PCR

Total RNA was extracted from tissues and Bel-7404 cells treated with DMSO or 40 μM Tan IIA for 24 h before harvest. The cDNA was synthesized using All-in-One cDNA Synthesis SuperMix regent (Bimake, #B24403). All qPCR analyses were performed in triplicate in a 20 μl mixture by using 2×SYBR Green qPCR Master Mix (Bimake, #B21202). The primers for GAPDH, YAP1, LATS1, LATS2, CTGF, SMAD7, and TGFβR1 have been reported in our previous studies [34]. Other primers used for qPCR are listed in Supplementary Table 3.

### Xenograft mouse model

In all, 5×10^6^ Bel-7404 cells expressing green fluorescent protein (GFP) empty vector or SMD7-Flag-GFP vector were subcutaneously injected into 6-week-old athymic nude mice (SLAC; Shanghai, China). After xenografts were visible, GFP control mice were divided into two groups, and then treated with DMSO or Tan IIA (10mg/kg/d) for another 20 days. Tumor size was measured every six days by using a caliper. GFP images were obtained using an *in vivo* imaging system for small animals, and the tumor volume was calculated using software. The mice were euthanized at 30 days after injection or 20 days after drug administration. All mouse experiments were performed according to the institutional guidelines of the Shanghai Municipal Hospital of Traditional Chinese Medicine.

### The *in vivo* imaging system for small animals

The xenograft mouse model expressed GFP *in vivo*. The mice were anesthetized and placed in an imaging camera box platform. The platform was controlled using software to an appropriate position, and the background image was obtained in the bright field. Next, the lights were turned off automatically, and then GFP images emitted in mice were photographed. The overlaid background maps obtained in bright field and dark field could visually show the location and intensity of specific regions in the mice.

### Bioinformatics

For SMAD7 co-expression network were obtained and analyzed from cBioPortal (https://www.cbioportal.org) and String (https://string-db.org) database. Genes co-expressed with SAMD7 were identified by conducting Gene Ontology biological function and Kyoto Encyclopedia of Genes and Genomes pathway analyses.

### Statistical analysis

Graphpad Prism 7.0 was used for data analyses. Differences between groups were analyzed using Student’s *t*-test, one-way analysis of variance, and χ^2^ test. Values with *p< 0.05, ** *p*< 0.01, and ***p< 0.001 were regarded as statistically significant. All results are expressed as the means ± standard deviation of three independent experiments.

## Supplementary Material

Supplementary Figures

Supplementary Tables
