# Supplementary material for: Tanshinone IIA mediates SMAD7-YAP interaction to inhibit liver cancer growth by inactivating the transforming growth factor beta signaling pathway
Source: Aging (Albany NY). 2019 Nov 11;11(21):9719–37. doi: 10.18632/aging.102420 (PMC6874425; doi:10.18632/aging.102420)
Supplement: Supplementary Figures [file aging-11-102420-s001.pdf]

SUPPLEMENTARY FIGURES

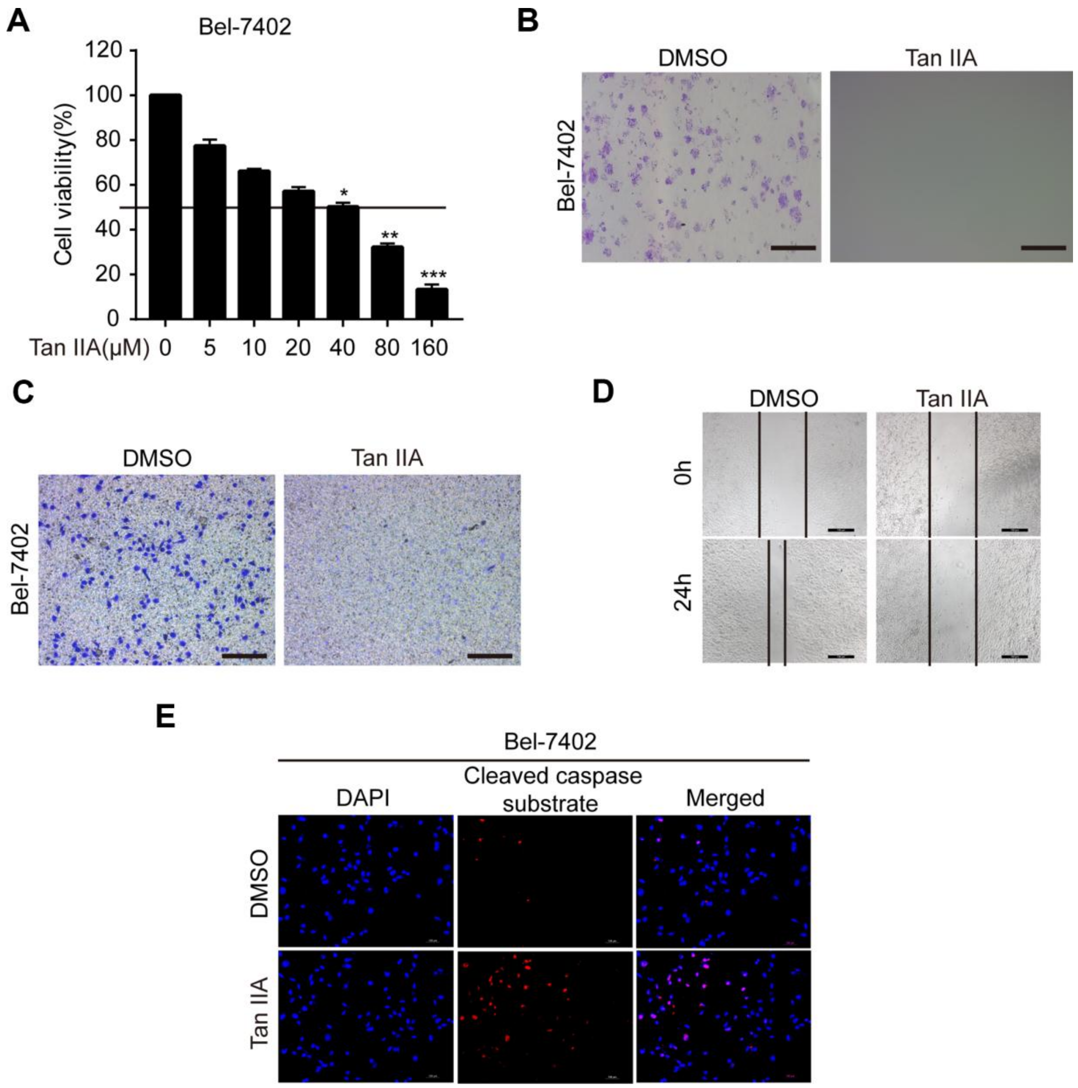

Supplementary Figure 1. (**A**) Cell viability of Bel-7402 cells treated with DMSO or dose dependent Tan IIA was determined by CCK-8 cytotoxicity test. *p<0.05, **p<0.01, ***p<0.001 vs DMSO. (**B**) Colony formation assay was to determine cell clonogenic ability in Bel-7402 cells with DMSO or 40 μM Tan IIA. Scale bars: 50 μm. (**C**) Cell invasion ability was measured by Transwell invasion assay in Bel-7402 cell lines treated with DMSO or 40 μM Tan IIA. Scale bars: 50 μm. (**D**) Cell wound healing assay was performed to measure cell migration ability in Bel-7402 cells treated with DMSO or 40 μM Tan IIA. The representative images were taken in different time points. Scale bars: 100 μm. (**E**) Tan IIA induced apoptosis marker cleaved caspase substrate expression measured by immunofluorescence assay in Bel-7402 cells. Scale bars: 100 μm.

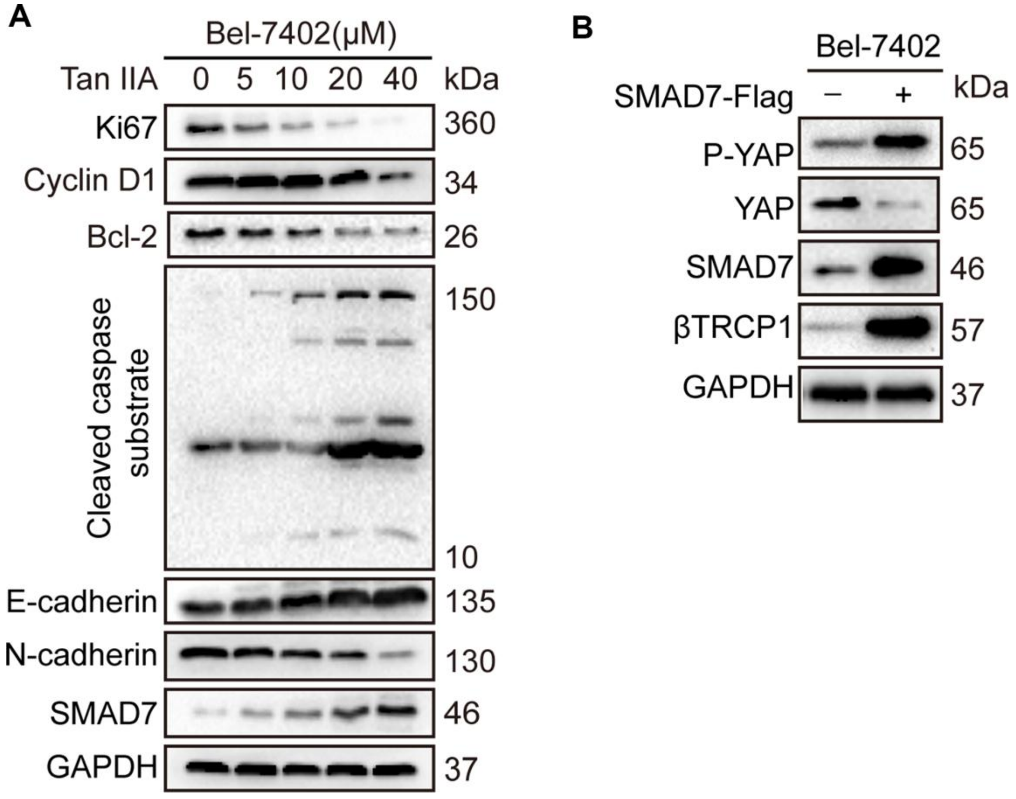

Supplementary Figure 2. (**A**) Bel-7402 cells were cultured with DMSO or dose dependent Tan IIA (5, 10, 20, 40 μM) for 24 h and protein expression levels of Ki67, CyclinD1, Bcl2, Cleaved Caspase Substrate, E-cadherin and N-cadherin were measured by western blot assay. (**B**) P-YAP, YAP and βTrcp1 protein expression level were measured by western blot assay with or without SMAD7-Flag overexpression in Bel-7402 cells.
