# Supplementary material for: Tanshinone IIA mediates SMAD7-YAP interaction to inhibit liver cancer growth by inactivating the transforming growth factor beta signaling pathway
Source: Aging (Albany NY). 2019 Nov 11;11(21):9719–37. doi: 10.18632/aging.102420 (PMC6874425; doi:10.18632/aging.102420)
Supplement: Supplementary Tables [file aging-11-102420-s002.pdf]

SUPPLEMENTARY TABLES

Supplementary Table 1. The biological significance gene lists.

Gene ID	P value	P value summary
**BIRC2**	0.0113	*
**CCND1**	0.0444	*
**CDKN1B**	0.006	**
**CFLAR**	0.032	*
**DLC1**	0.0305	*
**GADD45B**	0.0338	*
**IGFBP3**	0.0031	**
**ITGB1**	0.0409	*
**KDR**	0.0081	**
**PTGS2**	0.0055	**
**SMAD6**	0.0121	*
**SMAD7**	0.0018	**
**TGFA**	0.0385	*
**TGFB1**	0.0057	**
**TGFBR2**	0.001	**
**TNFRSF10B**	0.0058	**

Supplementary Table 2. Primers used for vectors.

Name	Sequence (5′-3′)
SMAD7-Flag (PGIPZ2a)-F	GAAAACCCTGGACCCATGGCTAGCATGGA TTACAAGGATGACGATGACAAGTTCAGGACCAAACGATCT
SMAD7-Flag (PGIPZ2a)-R	GGGAGAGGGGCGGAATTTGCGGCCGCCTACCGGCTGTTGAAGAT
SMAD7-sgRNA (LentiCrispr v2)-F	CACCGGGGAGCGAGTAGGACGAGGG
SMAD7-sgRNA (LentiCrispr v2)-R	AAACCCCTCGTCCTACTCGCTCCCC

Supplementary Table 3. Primers used for qPCR.

Gene	Sequence (5′-3′)
SMAD1-F	ACCTGCTTACCTGCCTCCTGAA
SMAD1-R	AACGCTTCACCCACACGATTG
SMAD2-F	ATGTCGTCCATCTTGCCATT
SMAD2-R	TCCATTCTGCTCTCCTCCAC
SMAD3-F	CCAGTGCTACCTCCAGTGTT
SMAD3-R	CTGGTGGTCGCTAGTTTCTC
SMAD4-F	GCTGCTGGAATTGGTGTTGATG
SMAD4-R	AGGTGTTTCTTTGATGCTCTGTCT
BIRC3-F	AAGCTACCTCTCAGCCTACTTT
BIRC3-R	CCACTGTTTTCTGTACCCGGA
